# Antibody Responses to Influenza A/H1N1pdm09 Virus After Pandemic and Seasonal Influenza Vaccination in Healthcare Workers: A 5-Year Follow-up Study

**DOI:** 10.1093/cid/ciy487

**Published:** 2018-06-09

**Authors:** Mai-Chi Trieu, Åsne Jul-Larsen, Marianne Sævik, Anders Madsen, Jane Kristin Nøstbakken, Fan Zhou, Steinar Skrede, Rebecca Jane Cox

**Affiliations:** 1 Influenza Centre, Department of Clinical Science, University of Bergen; 2 K. G. Jebsen Centre for Influenza Vaccine Research, Department of Clinical Science, University of Bergen, Bergen, Norway; 3 Division for Infectious Diseases, Department of Medicine, Haukeland University Hospital, Bergen, Norway; 4 Department of Clinical Science, University of Bergen, Bergen, Norway; 5 Department of Research and Development, Haukeland University Hospital, Bergen, Norway

**Keywords:** pandemic influenza vaccination, seasonal influenza vaccination, antibody, hemagglutination inhibition, healthcare worker

## Abstract

**Background:**

The 2009 influenza pandemic was caused by the A/H1N1pdm09 virus, which was subsequently included in the seasonal vaccine, up to 2016/2017, as the A/H1N1 strain. This provided a unique opportunity to investigate the antibody response to H1N1pdm09 over time.

**Methods:**

Healthcare workers (HCWs) were immunized with the AS03-adjuvanted H1N1pdm09 vaccine in 2009 (N = 250), and subsequently vaccinated with seasonal vaccines containing H1N1pdm09 for 4 seasons (repeated group), <4 seasons (occasional group), or no seasons (single group). Blood samples were collected pre and at 21 days and 3, 6, and 12 months after each vaccination, or annually (pre-season) from 2010 in the single group. The H1N1pdm09-specific antibodies were measured by the hemagglutination inhibition (HI) assay.

**Results:**

Pandemic vaccination robustly induced HI antibodies that persisted above the 50% protective threshold (HI titers ≥ 40) over 12 months post-vaccination. Previous seasonal vaccination and the duration of adverse events after the pandemic vaccination influenced the decision to vaccinate in subsequent seasons. During 2010/2011–2013/2014, antibodies were boosted after each seasonal vaccination, although no significant difference was observed between the repeated and occasional groups. In the single group without seasonal vaccination, 32% of HCWs seroconverted (≥4-fold increase in HI titers) during the 4 subsequent years, most of whom had HI titers <40 prior to seroconversion. When excluding these seroconverted HCWs, HI titers gradually declined from 12 to 60 months post–pandemic vaccination.

**Conclusions:**

Pandemic vaccination elicited durable antibodies, supporting the incorporation of adjuvant. Our findings support the current recommendation of annual influenza vaccination in HCWs.

**Clinical Trials Registration:**

NCT01003288.

## Teaser

Adjuvanted pandemic H1N1pdm09 vaccine induced durable antibody responses for up to 60 months post-vaccination in 44% of healthcare workers. Annual seasonal vaccination significantly boosted antibody responses. Without revaccination, individuals with hemagglutination inhibition titers <40 were more vulnerable to H1N1pdm09 infection.

Influenza is a major respiratory pathogen that causes annual epidemics/outbreaks and occasional pandemics. Annual and pandemic influenza vaccination are recommended for high-risk populations and occupational groups, including healthcare workers (HCWs), to mitigate the impact of influenza epidemics. However, the vaccination rates of HCWs remain low in many countries, especially in Norway [1].

In 2009, the first pandemic of the 21^st^ century was caused by the novel influenza A/H1N1 viruses (H1N1pdm09). Mass vaccination was the most effective prophylactic measure to prevent infection and limit viral spread in the community. In general, early vaccination of HCWs during a pandemic maintains the integrity of the healthcare system, reduces absenteeism, and prevents spread of the virus in the hospital [2]. During this pandemic, the use of the oil-in-water emulsion AS03-adjuvanted monovalent H1N1pdm09 vaccine allowed dose sparing, increasing the number of vaccine doses available. This vaccine had improved immunogenicity [3, 4] and vaccine effectiveness against laboratory-confirmed influenza infection and hospitalization [5].

In Norway, HCWs were among the first to receive the AS03-adjuvanted pandemic H1N1pdm09 vaccine, in October 2009, just before the peak of pandemic activity [6]. The same H1N1pdm09 strain was included in the seasonal vaccines as the A/H1N1 component from 2010/2011 to 2016/2017, while the A/H3N2 and B viruses in the vaccine were updated more frequently. This provided a unique opportunity to investigate the H1N1pdm09-specific vaccine-induced antibodies after a pandemic and subsequent annual vaccination.

We conducted a 5-year longitudinal study of 250 HCWs vaccinated with the AS03-adjvuanted H1N1pdm09 vaccine. Our findings provide new insight into the long-term antibody responses after a pandemic and susbequent annual vaccination, and have implications for influenza vaccination policies.

## MATERIALS AND METHODS

### Clinical Trial

**Figure 1.**
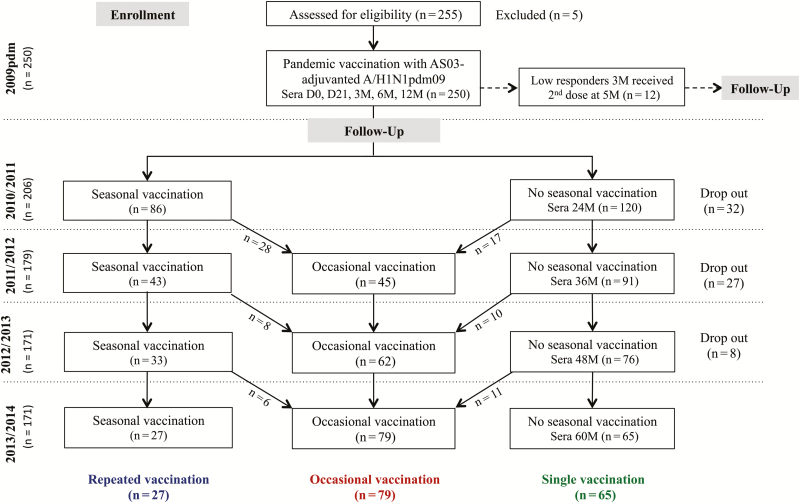
The study flow chart. Healthcare workers (HCWs) were recruited to an open-label 5-year extension of a single-arm clinical trial in 2009 (European Clinical Trials Database, EudraCT 2009-016456-43; www.clinicaltrials.gov, NCT01003288) [6]. All HCWs received a single dose of the AS03-adjuvanted monovalent pandemic A/H1N1pdm09 vaccine, except 12 low-responders, who were identified at 3 months post–pandemic vaccination and received a second dose at 5 months after the first dose. During 2010/2011–2013/2014, HCWs voluntarily received the trivalent seasonal inactivated vaccines, containing the A/H1N1pdm09 as the A/H1N1 component during the whole study period, while the A/H3N2 and B viruses changed between seasons. During the course of the study, HCWs did not always provide samples in each season, due to shift work, statutory leave (sick or parental leave), leaving employment at the hospital, or death. The drop out column shows the number of HCWs, irrespective of seasonal vaccination status, who dropped out in each season. At the end of the study, HCWs were divided into 3 groups based on their seasonal vaccination status: repeated (vaccination in all 4 seasons from 2010/2011–2013/2014), occasional (vaccination in <4 seasons), and single (no seasonal vaccination after pandemic vaccination in 2009).

HCWs (N = 250) were immunized with the AS03-adjuvanted pandemic H1N1pdm09 vaccine between October 2009 and March 2010 at Haukeland University Hospital, Norway. All HCWs provided written informed consent before inclusion in the study and also for the 4-year extension between 2010/2011–2013/2014 (Figure 1). The study was approved by the regional ethics committee (REKVest-2012/1772) and the Norwegian Medicines Agency (Clinical trials.gov NCT01003288) [6]. HCWs who had a virologically-confirmed H1N1pdm09 infection, an oral temperature >38°C in the preceding 72 hours, or an acute respiratory infection up to 7 days prior to immunization were excluded from the study.

### Diary Card

HCWs received a diary card to record the incidence and severity (mild, moderate, or severe) of local and/or systemic adverse events (AEs) in the 21 days post–pandemic vaccination [6]. The use of antipyretic/relief medication or anti-influenza drugs and the occurrence of intercurrent illnesses were also recorded.

### Vaccines

The 2009 pandemic vaccine was the AS03-adjuvanted split virus vaccine, containing 3.75 μg hemagglutinin of A/California/7/2009 (H1N1) (Pandemrix, GlaxoSmithKline-GSK, Belgium). The trivalent seasonal inactivated influenza vaccine (IIV; either subunit [Influvac, Abbott Laboratories] or split-virion [Vaxigrip, Sanofi Pasteur]) contained 15 μg hemagglutinin per strain and was used from 2010/2011 to 2013/2014. The A/H1N1 strain was A/California/07/2009(H1N1) throughout the study, although the A/H3N2 and B viruses changed between seasons.

### Sampling

Blood samples were collected pre–pandemic vaccination (day 0) and at 21 days and 3, 6, 12, 24, 36, 48, and 60 months post–pandemic vaccination in all HCWs. HCWs immunized with seasonal IIV provided additional blood samples at 21 days and 3 and 6 months after each vaccination. All serum samples were coded with a unique identification number, aliquoted, and stored at -80°C until analyzed.

### Hemagglutination Inhibition Assay

1 volume of serum was treated with 4 volumes of receptor- destroying enzyme (Seiken, Japan) at 37°C overnight before inactivation at 56°C for 30 minutes. Serial 2-fold dilutions of sera were tested in duplicate with 8 hemagglutinating units of A/California/07/2009(H1N1) (National Institute for Biological Standards and Control, UK, and International Reagent Resources, USA) and 0.7% (volume/volume) turkey red blood cells, as previously described [6]. The hemagglutination inhibition (HI) titer was read as the reciprocal of the highest dilution that inhibited 50% hemagglutination. The geometric mean HI titer (GMT) was calculated for each HCW, and titers <10 were assigned a value of 5 for calculation purposes. Sera with non-specific binding to turkey red blood cells or HI titers <40 were additionally treated by preadsorption with turkey red blood cells for 1 hour at 4°C with 30-minute mixing intervals, before reanalyzing.

### Statistical Analyses

Differences in demographics and clinical factors between the vaccination groups were examined by chi-square independence tests and adjusted in general linear models. HI data were log-transformed and evaluated in linear mixed-effect models for the annual effect of vaccination, or group differences, adjusted for repeated-measure subject variance and demographic factors. Post hoc tests were performed with Bonferroni correction. A correlation between HI titers and years post–pandemic vaccination was identified using linear regression models. *P* < .05 was considered statistically significant. All analyses were conducted in SPSS-Statistics version-24 (IBM Corporation, USA) and visualized in Prism version-7 (GraphPad Software, USA).

## RESULTS

### Study Population

HCWs (N = 250) were immunized with the adjuvanted pandemic vaccine in 2009 (78% females; mean age 40.4, range 21–67 years). During 2010/2011–2013/2014, the hospital recommended annual vaccination and provided the vaccine free of charge, but vaccination was voluntary. At the end of 5 years, HCWs were grouped by their seasonal vaccination status, either IIV for 4 seasons (repeated, n = 27), <4 seasons (occasional, n = 79), or no further seasons (single, n = 65; Figure 1). There were no significant differences in demographics between the 3 groups (Table 1), except that a history of previous IIV pre-2009 was more common in the repeated and occasional groups compared to the single group. In total, the study included 929 person-years and a total of 2562 serum samples.

**Table 1.** The Demographics and Clinical Characteristics of the Study Cohort

Characteristic^a^	All (N = 250)	Repeated (n = 27)	Occasional (n = 79)	Single (n = 65)	*P*-value^b^	Adjusted *P*-value^c^
Mean age ± SD	40.4 ± 11.9	44.9 ± 11.1	41.2 ± 11.8	40.0 ± 10.6	.103	.210
Gender					.777	.579
Female	195 (78.0)	23 (85.2)	63 (79.7)	54 (83.1)	-	-
Male	55 (22.0)	4 (14.8)	16 (20.3)	11 (16.9)	-	-
Working department					**.016**	.287
Infectious disease	44 (17.6)	9 (33.3)	18 (22.8)	6 (9.2)	-	-
Other clinical	107 (42.8)	8 (29.6)	41 (51.9)	31 (47.7)	-	-
Non-clinical	99 (39.6)	10 (37.0)	20 (25.3)	28 (43.1)	-	-
High-risk conditions^d^	26 (10.4)	2 (7.4)	8 (10.1)	5 (7.7)	.844	.955
Previous seasonal vaccination	148 (59.2)	23 (92.0)	55 (69.6)	24 (37.5)	**.000**	**.021**
Seasonal vaccination in 2009	44 (17.6)	5 (20.0)	11 (13.9)	7 (10.8)	.517	.871
Influenza illness during 2009					.548	.699
Yes	7 (3.0)	1 (4.0)	4 (5.3)	2 (3.3)	-	-
Possible	6 (2.6)	2 (8.0)	1 (1.3)	2 (3.3)	-	-
Adverse events after pandemic vaccination					.380	.766
No reaction	22/248 (8.9)	2 (7.7)	7 (8.9)	3 (4.6)	-	-
Only local reactions	83/248 (33.5)	12 (46.2)	27 (34.2)	22 (33.8)	-	-
Only systemic reactions	13/248 (5.2)	3 (11.5)	4 (5.1)	2 (3.1)	-	-
Both local and systemic reactions	130/248 (52.4)	9 (34.6)	41 (51.9)	38 (58.5)	-	-
Time to resolution of adverse events after pandemic vaccination					**.012**	.074
Within 2 days	129/226 (57.1)	21/24 (87.5)	42/72 (58.3)	29/62 (46.8)	-	-
Within 3–5 days	78/226 (34.5)	3/24 (12.5)	26/72 (36.1)	26/62 (41.9)	-	-
More than 5 days	19/226 (8.4)	0/24 (0.0)	4/72 (5.6)	7/62 (11.3)	-	-

All 250 participants received the AS03 adjuvanted pandemic vaccine in 2009. Groups were divided based on their vaccination history at the end of the 5 years of the study. During 2010/2011–2013/2014, healthcare workers who received seasonal vaccinations in all 4 seasons, less than 4 seasons, or none of the seasons were assigned to the repeated, occasional, or single groups, respectively. For further information, see Figure 1. Bold values show statistical significance.

Abbreviations: SD, standard deviation.

^a^Data were presented as number (%), unless otherwise specified.

^b^ *P*-value was determined by mean comparison between the 3 groups for age or chi-square independence test for other characteristics.

^c^Adjusted *P*-value was calculated using multivariate analyses in general linear model (R-square = 0.560).

^d^High-risk conditions included pregnancy, chronic respiratory diseases, neurological diseases, immunosuppressive diseases, heart diseases, diabetes, and obesity.

### Preexisting Antibodies Before Pandemic Vaccination

**Figure 2.**
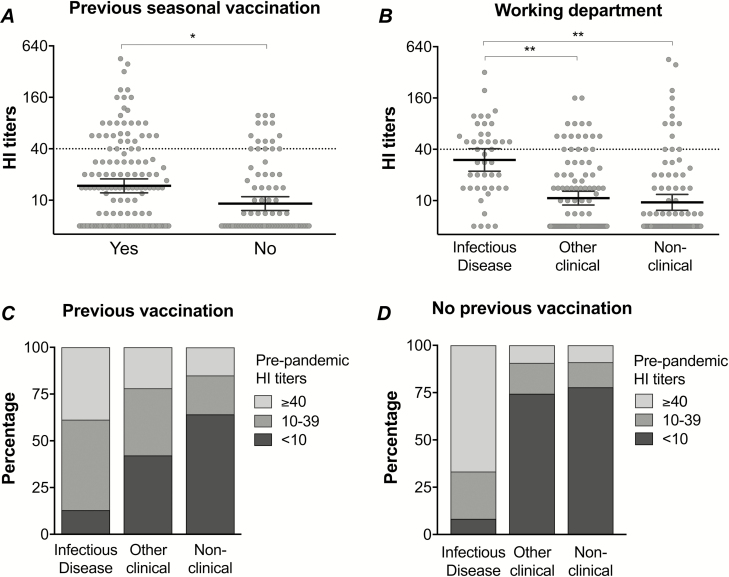
The preexisting H1N1pdm09-specific hemagglutination inhibition (HI) antibodies, prior to pandemic vaccination in 2009. The HI titers pre–2009 pandemic vaccination in healthcare workers (HCWs) are stratified by (*A*) previous seasonal vaccination status or (*B*) working department. Each symbol represents an individual HI titer, with the horizontal lines representing the geometric mean HI titer and 95% confidence interval. The dotted line indicates the protective threshold HI titer of 40. The percentage of HCWs from different working departments with a history of (*C*) previous seasonal vaccination or (*D*) no previous seasonal vaccination, stratified by HI antibody levels pre–2009 pandemic vaccination: HI titers <10 (undetectable antibodies), 10–39 (detectable antibodies), or ≥40 (protective antibodies). *P < .01. **P < .001.

HCWs with laboratory-confirmed H1N1pdm09 infection were excluded from the study; however, 52/250 (21%) of HCWs had H1N1pdm09-specific antibodies above the 50% protective threshold (HI titers ≥ 40) pre–pandemic vaccination. HCWs who were previously vaccinated with IIV before 2009 or worked on the infectious disease wards [6] had significantly higher HI titers pre–pandemic vaccination than those without a previous vaccination or those working at another clinical or non-clinical department (Figure 2).

Preexisting HI antibodies were more commonly found in HCWs working on infectious disease wards than other departments, regardless of previous IIV (Figure 2), suggesting an occupational exposure. Multivariate analysis showed that HI antibodies before pandemic vaccination were associated with work departments, rather than previous IIV (Supplementary Table 1). When considering detectable pre–pandemic vaccination HI antibodies (HI titers ≥ 10) as exposure to the pandemic virus, HCWs from the infectious diseases department were at either a 2.0- (95% confidence interval [CI] 1.6–2.5) or 2.9- (95% CI 2.1–4.0) fold higher risk of pandemic virus exposure than HCWs from other clinical or non-clinical departments, respectively.

### Immunogenicity of 2009 Adjuvanted Pandemic Vaccine

**Figure 3.**
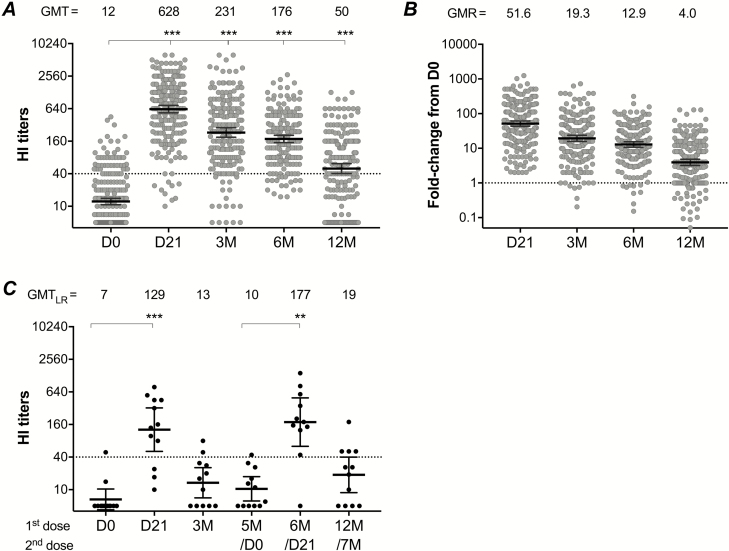
The H1N1pdm09-specific hemagglutination-inhibition (HI) antibody response after pandemic vaccination. *A* The HI titers of all vaccinated participants are shown pre-vaccination (D0) and at 21 days (D21) and 3, 6, and 12 months (3M, 6M, and 12M, respectively) post-vaccination during 2009. Each symbol represents an individual HI titer, with the horizontal lines representing the geometric mean HI titer (GMT) and 95% confidence interval (CI). The GMT is shown for each time point above the graph. The dotted line indicates the protective threshold HI titer of 40. *B* Fold-changes in HI titer from pre- (D0) to post–pandemic vaccination (D21, 3M, 6M or 12M) are shown. Each symbol represents an individual fold-change, with the horizontal lines representing the geometric mean ratio (GMR) and 95% CI, generated from all individual fold changes. The GMR is shown for each time point above the graph. The dotted line indicates the fold change of 1, meaning no change in HI titers between pre- and post-vaccination. *C* The HI titers in 12 low-responders (LRs), who received 2 doses of pandemic vaccine in the 2009 pandemic vaccination. These LRs had HI titers <40 or a <4-fold increase from pre-vaccination titers at 3 months post–pandemic vaccination, and received the second dose at 5 months. The HI titers of these 12 LRs were excluded from the 2009 data of all vaccinated participants at 6 and 12 months after the first dose. Pre- and post-vaccination HI titers of all participants were log-transformed and compared using linear mixed-effect models adjusted for subject variance with repeated measures and demographic factors. Post hoc tests were performed with Bonferroni correction. HI titers of LRs were compared using non-parametric repeated-measure Friedman tests. **P < .01. ***P < .001.

HI antibodies were robustly induced following the AS03-adjuvanted pandemic vaccination in 2009. The GMT peaked at 21 days and waned over 3, 6, and 12 months post-vaccination, although it persisted above the 50% protective threshold (HI titers ≥ 40; Figure 3). The pandemic vaccine fulfilled all 3 criteria of the European Committee for Medicinal Products for Human Use (CHMP), which were pre- and post-vaccination geometric mean ratio >2.5, seroconversion rate >40%, and seroprotection rate >70%, for up to 6 months [6]. At 12 months post–pandemic vaccination, 2 of the 3 CHMP criteria were fulfilled with the geometric mean ratio of 4 (Figure 3) and the seroconversion rate of 58.3%; however, the seroprotection rate was 63%, below the 70% criterion (Supplementary Table 2).

At 3 months post–pandemic vaccination, 30/206 (14.6%) HCWs were identified as low responders (LRs), with HI titers <40 or a <4-fold increase from pre-vaccination titers. All LRs were offered a second dose of the pandemic vaccine [7], and 12 LRs (67% female; mean age 37.3, range 23–63 years) chose to be revaccinated at 5 months after the first dose. Thus, HI responses from these 12 LRs were excluded from the 2009 6- and 12-month data. The HI antibodies in LRs were significantly boosted, to above the 50% protective threshold, at 21 days after the second dose; this result was comparable with the HI antibodies of other HCWs at the equivalent time point (6 months after the first dose), which then decreased to HI titer <40 at 7 months (Figure 3).

### Reactogenicity of 2009 Adjuvanted Pandemic Vaccine

**Figure 4.**
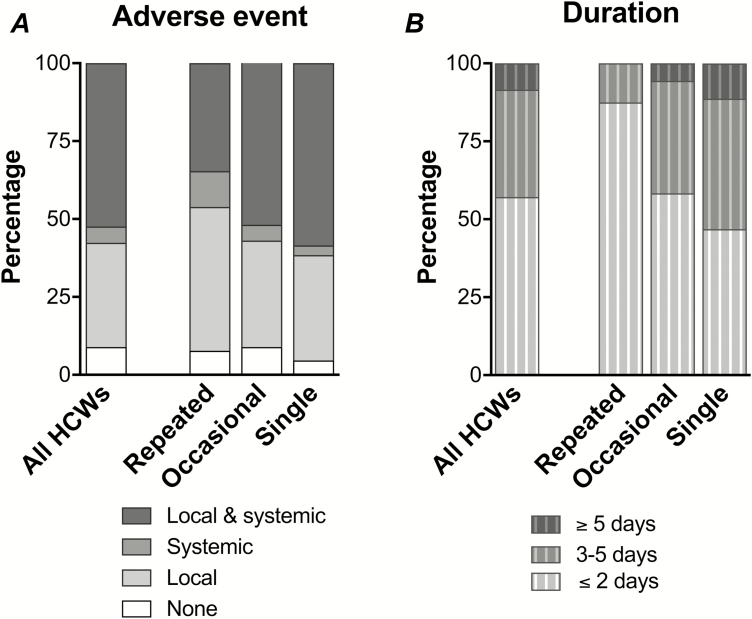
The adverse events (AEs) after pandemic vaccination. Healthcare workers (HCWs) were provided with a diary card where they self-recorded the incidence and duration of local (erythema/redness, itching, oedema/swelling, pain/tenderness at the injection site, ecchymosis, hardness/induration) and/or systemic (fever, malaise, shivering/chills, fatigue, headache, sweating, myalgia, arthralgia, respiratory symptoms, and diarrhea) AEs during the 21 days after pandemic vaccination. Only 2 HCWs (2/250) did not return their diary cards. *A* The percentage of HCWs reporting AE as none, local, systemic, or both local and systemic, stratified by the vaccination groups (repeated, occasional, or single group). *B* The percentage of HCWs reporting the incidence of any AE that resolved in durations of ≤2, 3–5, or ≥5 days, stratified by the vaccination groups.

After the 2009 pandemic vaccination, 226/248 (91%) of HCWs reported AEs, which were mainly mild to moderate reactions, with 6 severe AEs that required medication. No serious AEs that required hospitalization were reported [6]. Interestingly, we observed higher frequencies of both local and systemic AEs in the single group, whereas no or only local AEs were more common in the repeated group (Figure 4), although the difference was not statistically significant. The single group experienced a significantly longer duration of AEs (≥3 days) than the repeated and the occasional groups (≤2 days; Figure 4). However, multivariate analyses showed that a previous IIV before 2009, rather than an AE after the pandemic vaccination, significantly impacted upon the vaccination groups (Table 1), suggesting that the main influencing factor was a personal habit of annual vaccination.

### Persistence of Hemagglutination Inhibition Antibodies After Pandemic Vaccination and No Subsequent Seasonal Vaccination

**Figure 5.**
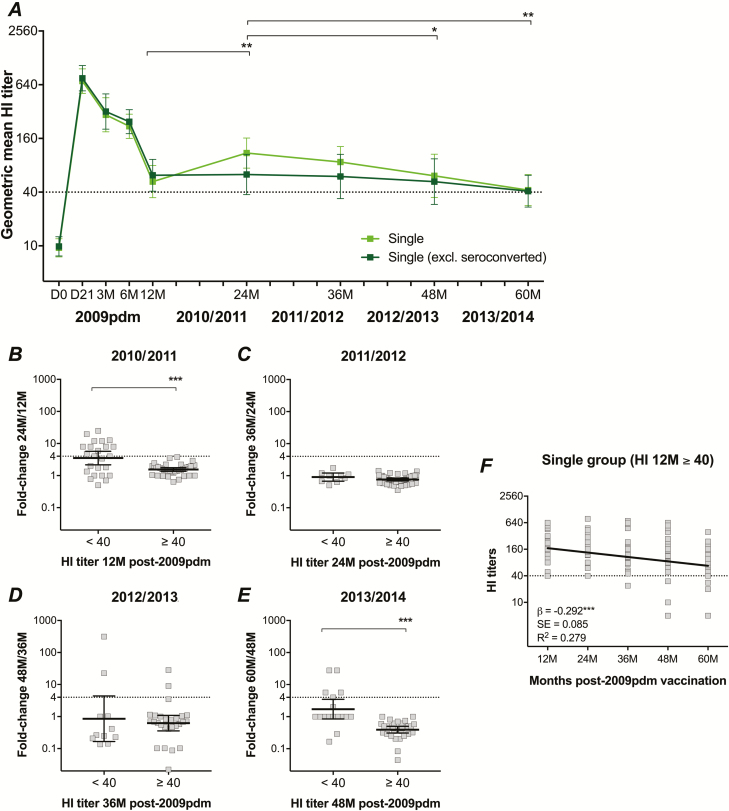
The H1N1pdm09-specific hemagglutination-inhibition (HI) antibody titers over 5 years after only pandemic vaccination. As the single group chose not to receive further vaccinations, data are only available after the pandemic vaccination (2009pdm) and prior to each influenza season (24, 36, 48, and 60 months post–pandemic vaccination: 24M, 36M, 48M, and 60M, respectively). A total of 21/65 healthcare workers (HCWs) seroconverted (>4-fold increase in HI titers) during 4 years of follow-up. *A* The geometric mean HI titers with 95% confidence interval over 5 years are presented, both in the single group and after excluding all HCWs who seroconverted (4-fold increase in titers). The dotted line indicates the protective threshold HI titer of 40. The fold-change in HI titers after 1 season in (*B*) 2010/2011, (*C*) 2011/2012, (*D*) 2012/2013, and (*E*) 2013/2014, stratified by HCWs with pre-season HI titers <40 or ≥40. *F* The decline of protective HI titers over time in the single group whose HI titers persisted ≥40 at 12 months (12M) post–pandemic vaccination. HI titers of the single group were log-transformed and compared between years using linear mixed-effect models adjusted for subject variance with repeated measures and demographic factors. Post hoc tests were performed with Bonferroni correction. Fold changes were compared using non-parametric Mann-Whitney tests. A negative correlation between HI titers and years post–pandemic vaccination was identified using linear regression models. *P < .05. **P < .01. ***P < .001.

During the 60-month follow-up of the single group with no susbsequent IIV, the GMT was boosted at 21 days after pandemic vaccination, waned throughout 3, 6, and 12 months, then increased significantly from 12 to 24 months (*P* = .003), and gradually declined from 24 to 60 months (Figure 5). The increased GMT at 24 months without revaccination indicated that exposure or infection from circulating H1N1pdm09 viruses during the 2010/2011 season may have occurred.

Among 62 HCWs in the single group who had paired samples at 12 and 24 months post–pandemic vaccination, 13 (21.0%) of them seroconverted (≥4 fold-increase HI titers; Figure 5). Interestingly, all HCWs who seroconverted at 24 months had HI titers <40 at 12 months. No HCWs seroconverted between 24 to 36 months (Figure 5). However, 4/38 (10.5%) and 5/48 (10.4%) HCWs seroconverted from 36 to 48 months and 48 to 60 months, respectively (Figure 5). During the 4-year follow-up of the single group, at least 21/65 (32.3%) of HCWs with paired samples seroconverted, and 1 of those participants seroconverted in 2 seasons. Importantly, 19/21 (90.5%) of HCWs who seroconverted had HI titers <40 in the previous season, suggesting that HCWs with HI antibodies below the 50% protective threshold were more likely to be infected from circulating H1N1pdm09 viruses. When excluding these HCWs who seroconverted from the single group, the GMT gradually decreased from 12 to 60 months post–pandemic vaccination, although it persisted above the protective threshold (Figure 5).

After a single pandemic vaccination, HI titers remained ≥40 without revaccination in 39/62 (62.9%) of HCWs at 12 months and in 27/62 (44%) of HCWs throughout 60 months. To investigate the persistence of protective antibodies in the single group while reducing the possibility of bias from influenza exposure or infection, only HCWs with HI titers ≥40 at 12 months were included in further analyses. We found a negative correlation between HI titers and years post–pandemic vaccination (β = -0.292, *P* < .001; Figure 5), demonstrating the slow rate of antibody decline over time (approximately -18% change per year).

### Persistent Antibody Responses After Repeated and Occasional Seasonal Vaccination

**Figure 6.**
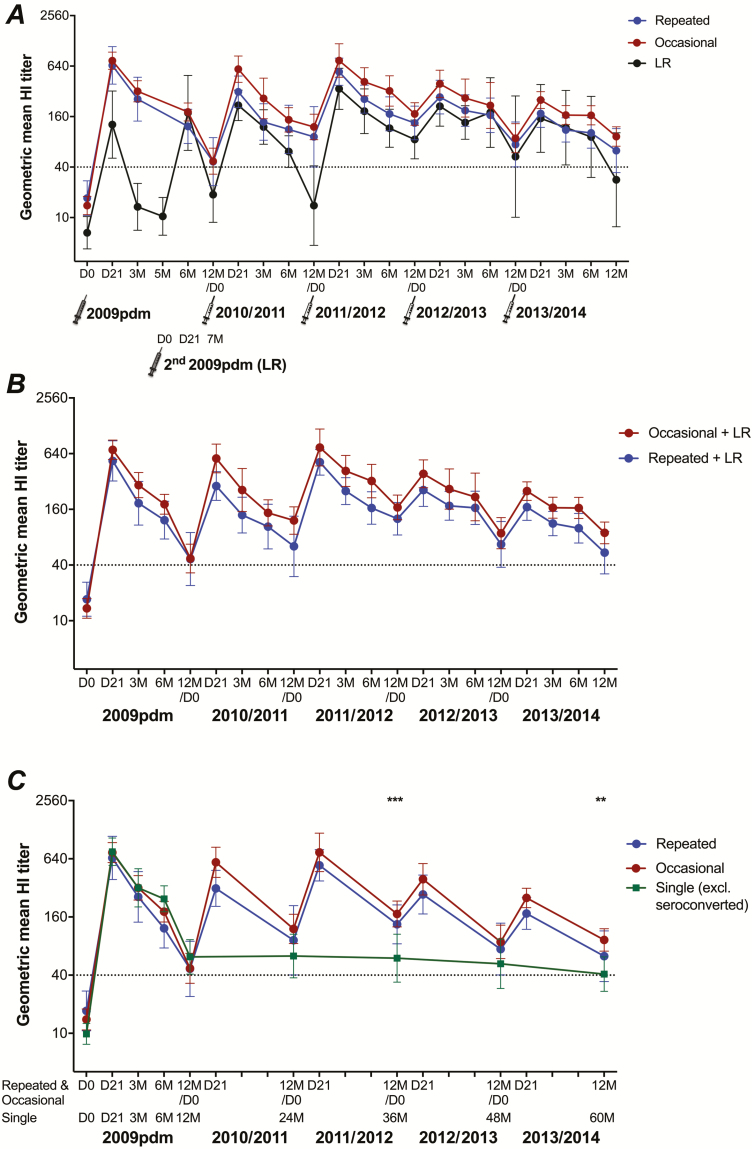
The 5-year dynamics of the H1N1pdm09-specific hemagglutination-inhibition (HI) antibody responses after pandemic vaccination and subsequent seasonal vaccinations. *A* The geometric mean HI titers with 95% confidence interval of vaccinated healthcare workers (HCWs) in the repeated group, the occasional group and in low responders (LRs) pre-vaccination (D0) and at 21 days (D21) and 3, 6, and 12 months (3M, 6M, and 12M, respectively) post-vaccination during the 2009 pandemic vaccination (2009pdm) through 2013/2014 are shown. In the 2009pdm, LRs received the second pandemic vaccination at 5 months (5M) and, therefore, their HI titers at 21 days and 7 months (7M) after the second vaccination are equivalent to the 6 and 12 month measures after the single pandemic vaccination in other participants. For each year/season, the HI titers at 12 months in the former year/season were considered as the pre-vaccination responses only for the HCWs who were vaccinated in the relevant next season. The dotted line indicates the protective threshold HI titer of 40. *B* The antibody responses of the repeated and occasional groups, including LRs who were either repeatedly vaccinated with the seasonal vaccine (LR repeated group, n = 6) or occasionally vaccinated (LR occasional group, n = 2). For more information on LRs, see Supplementary Figure 2. *C* The antibody responses of the repeated and occasional groups in comparison with the single group, excluding 21/65 HCWs who seroconverted (>4 fold-increase HI titers) during 2010/2011–2013/2014, at equivalent time points (12 months post–seasonal vaccination or prior to influenza season). HI antibody titers were log-transformed and compared between groups using linear mixed-effect models adjusted for subject variance with repeated measures and demographic factors. Post hoc tests were performed with Bonferroni correction. **P < .01, ***P < .001.

During the 2010/2011–2013/2014 seasons, HI antibodies were significantly boosted at 21 days after each IIV, and gradually waned over 3, 6, and 12 months post-vaccination, although they persisted above the HI titer of 40 in both the repeated and the occasional groups (Figure 6). Antibody responses and geometric mean ratios post-IIV during the 4 seasons were not significantly different between the repeated and occasional groups (Supplementary Table 3), although slightly higher HI titers were always observed in the occasional group. Furthermore, no significant differences were detected in HI titers post-IIV between HCWs who had and HCWs who had not been previously vaccinated before 2009 (Supplementary Figure 1). Interestingly, the pre-vaccinaton GMT at the start of each season from 2010/2011–2013/2014 remained ≥40, and was significantly higher than the GMT prior to the 2009 pandemic vaccination in both the repeated (*P* < .01) and the occasional (*P* < .001) groups.

Among 12 LRs who received 2 doses of the AS03-adjuvanted pandemic vaccine in 2009, 6 persons were annually vaccinated with IIV in the 4 subsequent seasons (repeated), 2 were occasionally vaccinated (occasional), and 1 chose not to receive IIV (single; Supplementary Figure 2). Lower GMTs post-IIV were observed in the 2010/2011 and 2011/2012 seasons in the vaccinated LRs, although they were not significant compared to the repeated and the occasional groups (Figure 6). During the 2012/2013 and 2013/2014 seasons, the antibody responses in LRs were comparable to the other groups. A similar trend in antibody responses between the repeated and the occasional groups was observed, with no significant difference when including the appropriate LRs (Figure 6).

Importantly, GMTs in the repeated and occasional groups were higher than in the single group (excluding seroconverted individuals) during the 4 post-pandemic seasons, although only significant at 12 months post-IIV in seasons 2011/2012 and 2013/2014 (equivalent to 36 and 60 months after pandemic vaccination; Figure 6).

## DISCUSSION

During the Norwegian mass vaccination campaign, HCWs were prioritized for the first rounds of vaccination to protect the integrity of the health system. Vaccination commenced 2 weeks prior to the peak of pandemic activity. From 2009, the H1N1pdm09 virus continued to circulate, replacing previous H1N1 strains, and was included in the seasonal vaccines up to the 2016/2017 season, providing a unique opportunity to study the long-term H1N1pdm09-specific antibody responses. This 5-year follow-up of antibody responses to the H1N1pdm09 virus after immunization with an AS03-adjuvanted pandemic vaccine provides new insight into the durability of the antibody response after pandemic vaccination and subsequent annual vaccinations. To our knowledge, this is the longest follow-up study after an adjuvanted vaccination.

During the pandemic, the use of the AS03-adjuvanted H1N1pdm09 vaccine allowed dose sparing to a quarter of the amount of antigen used in seasonal vaccines, and increased the immunogenicity of the vaccine [3, 4]. In our study, we found rapid and durable antibody responses, with all CHMP criteria fulfilled up to 6 months post–pandemic vaccination [6] and two-thirds of CHMP criteria fulfilled at 12 months. Remarkably, 1 dose of pandemic vaccine induced durable protective antibody titers throughout 60 months, without revaccination with IIV, in 44% (27/62) of HCWs in the single group. This was probably due to the AS03-adjuvant, which effectively activated innate immunity, increasing antigen uptake and presentation in the local draining lymph nodes [4] and leading to higher T- and B-cell responses than after immunization with non-adjuvanted vaccines [8–10]. The adjuvant elicits particularly good humoral responses by stimulating increased activation of naïve B cells and increasing the adaptability of recalled memory B cells, leading to the fine-tuning of the lineage specificity through further rounds of affinity maturation [8]. Our study has implications for future use of the AS03-adjuvant in pandemic or seasonal influenza vaccines and in other antibody-inducing vaccines.

Although the antibody persistence in our single group may be due to exposure/infection with circulating viruses, it is unlikely that all HCWs, especially those with HI titers ≥40, experienced clinical/subclinical infection during the study. The serological evidence of infection among 32% of the single group, the gradual decrease in antibody titers post–pandemic vaccination up to 2013/2014, and the declining protection over time since the latest vaccination [11] indicate that revaccination is required for improved protection.

Annual influenza vaccination is recommended by our hospital, although it is voluntary. HCWs in this study could, therefore, be divided according to their vaccination status of seasonal vaccinations for 4 years (repeated) and <4 years (occasional). Despite over half a century of seasonal influenza vaccination use, the long-term impact of annual vaccinations on antibody responses remains unclear. We found that H1N1pdm09-specific HI antibodies were boosted after each IIV, and the seroprotective titers were maintained over 12 months post-IIV in both the repeated and the occasional groups. The lack of significant differences in HI antibodies between these 2 groups suggests that repeated vaccination with the same H1N1pdm09 virus was not better than occasional vaccination. However, other immunological parameters, such as the quality of antibodies or memory B- and T-cells, which were not accessed here, have been shown to be improved after repeated vaccination [12–16]. Others have found that repeated vaccination did not lower protection [17, 18] and was beneficial at reducing viral shedding and infection, similarly to first-time vaccinees [19]. Furthermore, as the A/H3N2 and B strains, which are also included in the seasonal vaccine, undergo more frequent antigenic drift, it is important to have immunity against these more-frequently changing viruses. Therefore, we support the continuation of annual seasonal vaccination, even with the same vaccine antigen, to provide better protection in HCWs.

HCWs are at a significantly higher risk of influenza exposure and infection compared to adults working in other sectors [20]. We found that 21% of HCWs had detectable antibodies to H1N1pdm09 prior to pandemic vaccination, suggesting considerable exposure or asymptomatic subclinical infection. Indeed, as previously reported [6], compared to other departments in the hospital, working on the infectious disease wards, where infected patients were treated, was a risk factor for detectable preexisting H1N1pdm09 antibodies. These findings show the risk of influenza infection in HCWs who choose not to be vaccinated and who may, therefore, pose a risk of influenza transmission to patients and other HCWs. Furthermore, higher pre–pandemic vaccination H1N1pdm09-specific antibodies were observed in HCWs who had previous seasonal vaccinations compared to those never before vaccinated. In meta-analyses, pre-2009 IIV provided moderate cross-protection before the pandemic vaccine was available [21]. Our data show that HCWs who had a previous habit of seasonal vaccinations were more likely to continue to choose vaccination. Worryingly, only 33/250 (13%, including 6 LRs) of HCWs were annually vaccinated over the 5 years of this study. There is an urgent need to improve vaccination uptake, particularly in frontline HCWs, and this requires effective strategies to initiate a habit of annual vaccination in HCWs. In the future, the next generation of influenza vaccines should provide robust antibodies and long-lasting protection, while not causing prolonged AEs to overcome the requirement of annual vaccination.

HCWs are an important target group for influenza vaccination, as well as for well-kept records of influenza vaccination and self-reported influenza-like illnesses. We faced problems inherent in longitudinal 5-year studies, including losing participants due to long-term follow-up, change of vaccination status, missing samples, and no active virologic/influenza-like illness surveillance. A major caveat to this study is that antibody responses were assessed after the initial vaccination with an adjuvanted pandemic vaccine and subsequent repeated or occasional seasonal vaccination; therefore, a difference may be found in populations immunized with a non-adjuvanted pandemic vaccine. Further studies are required to evaluate immune responses against other viruses included in the seasonal IIV, as well as in high-risk groups recommended for annual vaccination.

In conclusion, 1 dose of adjuvanted pandemic vaccine induced robust, durable antibodies, supporting the use of adjuvanted influenza vaccines. Seasonal vaccination, whether repeated or occasional, boosted antibody responses and maintained the protective antibody levels. Without revaccination, HCWs with non-protective antibody titers are more likely to be infected with circulating viruses. Our study supports the continued policy of annual influenza vaccination of HCWs.

## Supplementary Data

Supplementary materials are available at *Clinical Infectious Diseases* online. Consisting of data provided by the authors to benefit the reader, the posted materials are not copyedited and are the sole responsibility of the authors, so questions or comments should be addressed to the corresponding author.

Supplementary Material

## Notes

***Acknowledgments.*** The authors thank all the healthcare workers who participated in this study, and staff at the Influenza Centre and the Bergen Clinical Vaccine Consortium for help with the study. We are grateful to the biostatisticians; Haukeland University Hospital, Bergen, Norway; and Dr Amit Bansal, University of Bergen, for helpful discussions and statistical advice.

***Financial support.*** This work was supported by intramural funding by the Influenza Centre at the University of Bergen and the Bergen Clinical Vaccine Consortium. The Influenza Centre is funded by the Ministry of Health and Care Services, Norway; the Research Council of Norway Globvac program (220670/H10); the European Union (Univax 601738, EU IMI115672FLUCOP, and EuroNanoMed2 271160); Helse Vest; and the K.G. Jebsen Centre for Influenza Vaccine Research. The authors confirm full access to all the data in the study and have final responsibility for the decision to submit for publication.

***Potential conflicts of interest.*** All authors: No reported conflicts. All authors have submitted the ICMJE Form for Disclosure of Potential Conflicts of Interest. Conflicts that the editors consider relevant to the content of the manuscript have been disclosed.
