# Supplementary material for: Antibody Responses to Influenza A/H1N1pdm09 Virus After Pandemic and Seasonal Influenza Vaccination in Healthcare Workers: A 5-Year Follow-up Study
Source: Clin Infect Dis. 2018 Jun 9;68(3):382–92. doi: 10.1093/cid/ciy487 (PMC6336911; doi:10.1093/cid/ciy487)
Supplement: Supplementary Material [file ciy487_suppl_supplementary_material.docx]

Antibody responses to influenza A/H1N1pdm09 virus after pandemic and seasonal influenza vaccination in healthcare workers: a five-year follow-up study

Mai-Chi Trieu,^1,2^ Åsne Jul-Larsen,^1,2^ Marianne Sævik,^3^ Anders Madsen,^1^ Jane Kristin Nøstbakken,^1^ Fan Zhou,^1,2^ Steinar Skrede,^3,4^ Rebecca Jane Cox^1,2,5^

**Supplementary files**

Supplementary Table 1

Supplementary Table 2

Supplementary Table 3

Supplementary Figure 1

Supplementary Figure 2

**Supplementary Table 1: Demographic and clinical characteristics of healthcare workers (HCW) with different levels of pre-pandemic vaccination antibodies.** The pre-pandemic vaccination A/H1N1pdm09-specific antibodies were assessed in the hemagglutination inhibition (HI) assay. The protective threshold was defined as HI titers ≥40. Pre-pandemic vaccination HI titers were divided into 3 levels: <10 (undetectable antibodies, below the limit of the assay), 10-39 (detectable antibodies), and ≥40 (protective antibodies). The characteristics of participants with different pre-vaccination HI titers were examined using compare-mean or chi-square tests then adjusted in general linear models.

| Characteristic* | Pre-pandemic vaccination HI titers | | | P-value^a^ | Adjusted p-value^b^ |
| --- | --- | --- | --- | --- | --- |
|  | <10  (N=133) | 10-39  (N=65) | ≥40  (N=52) |  |  |
| Age (mean ± SD) | 41.7 ± 11.9 | 41.3 ± 12.5 | 35.9 ± 9.7 | **0.008** | 0.992 |
| Sex |  |  |  | **0.024** | 0.609 |
| Female | 101 (51.8) | 58 (29.7) | 36 (18.5) | - | - |
| Male | 32 (58.2) | 7 (12.7) | 16 (29.1) | - | - |
| Working department |  |  |  | **0.000** | **0.003** |
| Infectious disease | 5 (11.6) | 18 (42.2) | 20 (46.5) | - | - |
| Other clinical | 59 (55.1) | 30 (28.0) | 18 (16.8) | - | - |
| Non-clinical | 69 (70.4) | 17 (17.4) | 12 (12.2) | - | - |
| High-risk conditions^c^ |  |  |  | 0.887 | 0.174 |
| Yes | 15 (57.7) | 6 (23.1) | 5 (19.2) | - | - |
| No | 118 (52.7) | 59 (26.3) | 47 (21.0) | - | - |
| Previous seasonal vaccination before 2009 | | | | **0.001** | 0.081 |
| Yes | 65 (43.9) | 49 (33.1) | 34 (23.0) | - | - |
| No | 67 (67.6) | 16 (16.2) | 16 (16.2) | - | - |

*Data was presented as number (%), if otherwise specified.

^a^P-value was determined by compare-mean for age and chi-square tests for other characteristics.

^b^Adjusted p-value was calculated using multivariate analysis in general linear models (R-square = 0.759)

^c^High-risk conditions include pregnancy, chronic respiratory diseases, neurological diseases, immunosuppressive diseases, heart diseases, diabetes, and obesity.

**Supplementary Table 2: Evaluation of the 2009 AS03-adjuvanted monovalent A/H1N1pdm09 pandemic vaccine according to the European Committee for Medicinal Products for Human Use (CHMP) criteria**. The evaluation was based on the vaccinees’ antibody responses against A/H1N1pdm09 assessed using the hemagglutination inhibition (HI) assay. Healthcare workers (HCWs) were vaccinated with a single dose of the AS03-adjuvanted pandemic vaccine in 2009 (N=250). Twelves HCWs, who were identified as low-responsders (LRs) at 3 months, received a second dose of the pandemic vaccine at 5 months. The antibody responses from LRs were therefore excluded from the evaluation of a single dose of pandemic vaccine at 6 and 12 months. The geometric mean titer (GMT) of all vaccinated HCWs are shown. Fold-change between pre- and post-vaccination were calculated for each participant and the pre- and post-vaccination geometric mean ratio (GMR) were generated from all individual fold-changes at each time point. The seroconversion rate is the percentage of vaccinees with >4 fold-increase in HI titers post-vaccination. Seroprotection rate is the percentage of vaccinees with HI titers above the 50% protective threshold (HI titers ≥40). For pandemic vaccines, all three of the CHMP criteria have to be met, which are GMR >2.5, seroconversion rate >40% and seroprotection rate >70%. These HCWs (n=207) were previoulsy analysed up to 3 months post-pandemic vaccination[6], where HI titers were standardised according to a conversion factor (0.6409) using the human post-pandemic H1N1 2009 infection plasma standard (09/194, produced by the National Institute of Biological Standards and Control, UK).

| Pandemic vaccination | n | GMT | GMR | Seroconversion rate (%) | Seroprotection rate (%) | No. of criteria met |
| --- | --- | --- | --- | --- | --- | --- |
| Day 0 | 250 | 12 | - | - | 20.8 | - |
| 21 days | 247 | 628 | 51.6 | 95.1 | 96.4 | 3/3 |
| 3 months | 206 | 231 | 19.3 | 89.3 | 92.2 | 3/3 |
| 6 months | 200 | 176 | 12.9 | 86.0 | 91.5 | 3/3 |
| 12 months | 211 | 50 | 4.0 | 58.3 | 63.2 | 2/3 |

**Supplementary Table 3: The geometric mean ratio (GMR) between pre- and 21-day post-seasonal vaccination of repeated and occasional groups during 4 post-pandemic seasons 2010/11 - 2013/14.** The fold-change in HI titers between pre- and 21-day post-vaccination was calculated for each subject in the repeated or occasional group for each season. Geometric mean of fold-change was generated from individual response pre- and post-vaccination HI titers and is presented as GMR. The GMR was calculated for the repeated or occasional group including low-responders (LR) who received 2 doses of the AS03-adjuvanted pandemic vaccine in 2009. No significant difference in GMR between repeated and occasional groups, with or without LR, was found using a linear mixed-effect model, adjusted for subject variance with repeated measures and demographic factors.

| Group | GMR D21 | | | |
| --- | --- | --- | --- | --- |
|  | **2010/11** | **2011/12** | **2012/13** | **2013/14** |
| Repeated (+ LR) | 7.5 (6.9) | 8.6 (10.8) | 1.9 (1.9) | 2.2 (2.4) |
| Occasional (+ LR) | 9.6 (9.3) | 4.7 (4.7) | 1.5 (1.5) | 2.2 (2.2) |

**Supplementary Figure 1: The 5-year dynamics of the H1N1pdm09-specific hemagglutination-inhibition (HI) antibody responses stratified by previous seasonal vaccination status.** The geometric mean HI titers (GMT) with 95% confidence interval (CI) of participants who had either previously received seasonal influenza vaccination or no vaccination before 2009 and subsequently either repeatedly or occasionally vaccinated with seasonal vaccines during seasons 2010/11-2013/14. There were no participants who had no previous seasonal vaccination before 2009 and subsequently repeatedly vaccinated with seasonal vaccines. The dotted line indicates the protective threshold HI titer of 40. No significant differences in antibody responses following pandemic and seasonal vaccinations were found using a linear mixed-effect model, adjusted for subject variance with repeated measures and demographic factors.

**Supplementary Figure 2: The flow chart of 5-year follow-up the low-responders (LRs).** In 2009, all healthcare workers (HCWs) received a single dose of the AS03-adjuvanted monovalent pandemic H1N1pdm09 vaccine. At 3-months post-pandemic vaccination, HCWs who had hemagglutination-inhibition (HI) antibody titers <40 or <4 fold-increase from pre-vaccination titers were identified as LRs and offered a second dose of the AS03-adjuvanted pandemic vaccine. Twelve HCWs received the second dose at 5-months. During 2010/11-2013/14, HCWs voluntarily received the trivalent seasonal inactivated vaccines containing the H1N1pdm09 as the A/H1N1 component during the whole study period, while A/H3N2 and B viruses changed between seasons. The drop out shows the number of HCWs irrespective of seasonal vaccination status who dropped out in each season.
